# Metabolic syndrome in patients with type 2 diabetes mellitus at Adama Hospital Medical College, Ethiopia: a hospital-based cross-sectional study

**DOI:** 10.3389/fcdhc.2023.1165015

**Published:** 2023-06-15

**Authors:** Tesfaye Getachew Charkos, Menberu Getnet

**Affiliations:** ^1^ School of Public Health, Adama Hospital Medical College, Adama, Ethiopia; ^2^ Department of Internal Medicine, Adama Hospital Medical College, Adama, Ethiopia

**Keywords:** metabolic syndrome, diabetes mellitus, risk factors, magnitude, Ethiopia

## Abstract

**Background:**

Metabolic syndrome is one of the most serious global public health problems. It is associated with a higher risk of heart attack and other cardiovascular diseases. However, the magnitude of metabolic syndrome among patients with type 2 diabetes mellitus is not well understood, especially in developing countries such as Ethiopia.

**Objective:**

To determine the magnitude of metabolic syndrome and associated factors among type 2 diabetes mellitus patients at Adama Hospital Medical College, Ethiopia, in 2022.

**Method:**

A facility-based cross-sectional study was conducted from September 1 to October 30, 2022. The data was collected through a self-administered questionnaire. A systematic random sampling method was used to select the participants. Data were entered using Epi Info version 7.2 and analyzed by SPSS version 23. Multivariable logistic regression was used to model this study. Statistical significance was set at p-values of < 0.05.

**Result:**

A total of 237 participants were included in this study, with a response rate of 95.1%. Overall, the magnitude of metabolic syndrome was 53.2% (95% CI: 46.8 - 59.6), 41.3% (95% CI: 35.0 - 47.5), and 41.8% (95% CI: 35.5 – 48.1) based on 2009 harmonized criteria of MetS, Revised National Cholesterol Education Program Adult Treatment Panel III (NCEP-ATP III), and International Diabetes Federation (IDF) criteria, respectively. In multivariable logistic analysis, urban residence (AOR=3.07, 95% CI: 1.46-6.42), earning a high income (AOR=5.87 95% CI: 1.8-19.1), history of cardiac illness (AOR=3.33, 95% CI: 1.41-7.84), history of hypertension (AOR=2.65, 95% CI: 1.22-5.78), dyslipidemia (AOR=4.47, 95% CI: 1.96-10.19), current cigarette smoker (AOR=6.2, 95% CI: 1.7-22.93), sedentary activity (AOR=3.62, 95% CI: 1.68-7.82), use of palm oil (AOR=4.87, 95% CI: 2.06-11.51), and BMI ≥25 kg/m^2^ (AOR=3.36, 95% CI: 1.57-7.16) were significantly associated with metabolic syndrome.

**Conclusion:**

The findings of this study suggested that the magnitude of metabolic syndrome among T2DM patients was high. We found consistent results using the NCEP-ATP III and IDF criteria. Similarly, urban residence, high income, history of cardiac, history of hypertension, dyslipidemia, current cigarette smoker, sedentary activity, palm oil, and BMI ≥25 kg/m^2^ were significantly associated with metabolic syndrome.

## Introduction

Metabolic syndrome (MetS) is a group of conditions that together raise the risk of coronary heart disease, diabetes, stroke, and other serious health problems, also known as insulin resistance syndrome (1). It is defined as the presence of at least three of the following five risk factors: triglyceride (TG) of > 150 milligrams per deciliter (mg/dl), high-density lipoprotein (HDL) of < 40 mg/dl for men and < 50 mg/dl for women, blood pressure (BP) of > 130/85mmHg, fasting blood glucose (FBG) of > 100 mg/dl, and waist circumference (WC) of >102 cm for men and > 88 cm for women.

Recently, MetS has become a serious global public health problem. MetS increases the risk of cardiovascular disease (CVD), type 2 diabetes mellitus (T2DM), stroke, and cardiovascular mortality in the general population (2). It was highly prevalent, ranging from 10% to 84% depending on the region, urban or rural environment, composition (sex, age, race, and ethnicity) of the population studied, and the definition of the syndrome used (3, 4). A recent meta-analysis revealed that the prevalence of MetS was 18.0%, 17.1%, and 11.1% based on the International Diabetes Federation (IDF), Revised National Cholesterol Education Program Adult Treatment Panel III (NCEP-ATP III), and World Health Organization (WHO) criteria, respectively (5). Similarly, the findings from a systematic review and meta-analysis indicated that the prevalence of MetS among T2DM patients in sub-Saharan African countries was 59.62% (4).

Several studies have inconsistently reported the prevalence of MetS in Ethiopia (6–8). The findings from the cross-sectional studies indicate that the prevalence of MetS ranged from 9.6% in rural areas to 12.5% in the capital city of Addis Ababa (6, 9). However, local and abroad studies have reported that the prevalence of MetS among patients with T2DM was significantly higher than the ordinary society. Jemere et al. found that MetS was prevalent among 56% of patients with T2DM and 44% of patients with hypertension (8) in Ethiopia. Even though several studies have been conducted in Ethiopia the reported results are still sparse. This may be due to the following reasons. First, all of the previous studies were based on cross-sectional study design. The data for this study design were collected at a point in time (a snapshot study). As a result, the findings may be overestimated or underestimated. Second, some of the previous studies involved a small sample size, which may lead to imprecise results. Last, different study settings may have also an impact on a small-scale study. Having taken all this into consideration, we aimed to determine the prevalence of MetS and its associated factors among patients with T2DM at Adama Hospital Medical College. To the best of our knowledge, our findings may add information to the existing literature since we used different study settings.

## Materials and methods

### Study area and design

A facility-based cross-sectional study was conducted from 1 March to 30 April 2022, at Adama Hospital Medical College, Ethiopia. Adama is 98 km east of Addis Ababa, in the East Shewa Zone, and its population size is 220,212 based on figures from the Central Statistical Agency in 2007. The population is made up of multiple ethnic groups and mixed cultures. Adama Hospital Medical College has a total of 184 beds and its admission rate was 173 patients per week. Its average outpatient flow was 856 and its annual outpatient flow is 226,000.

The source population was all patients with T2DM who had a follow-up at Adama Hospital Medical College. The participants were screened based on the following criteria: 1) type 2 diabetes mellitus patients, 2) aged 18 years or older, 3) able to give consent to participate in the study, and 4) capable of understanding and answering the questions. The participants that did not fulfill the inclusion criteria were excluded from the analysis. The study participants were selected using a systematic random sampling method.

### Study variables and measurements

The dependent variable was metabolic syndrome and the independent variables, including socio-demographic factors (sex, age, religion, body weight, height, place of residence, education level, and family income), behavioral factors (physical exercise, cigarette smoking, alcohol drinking, and khat chewing), health-related factors (diabetes duration, anti-diabetic drug, drug adherence, mental illness, and sleep disturbance), and dietary factors (dietary plan and diet type), were collected using a structured questionnaire. Anthropometric measurements of weight and height were measured using a seca^®^ weighing scale and stadiometer respectively, with light clothing and without shoes. Body mass index (BMI) was calculated as body weight (kg) divided by the squaring of body height (m^2^). Waist circumference is the measurement taken around the abdomen at the level of the umbilicus (belly button) in centimeters (cm). Two blood pressure measurements were taken 10 min apart for each participant using a standard adult mercury-based sphygmomanometer with the correct size arm cuff.

MetS was identified if at least 3 of the following 5 risk factors were fulfilled based on the IDF definition, 2009 harmonized criteria, and the NCEP-ATP III.

#### The revised National Cholesterol Education Program: Third Adult Treatment Panel

MetS is defined as having at least three of the following five risk factors: a WC of >102cm for men and >88 cm for women, BP of >130/85 mmHg or on treatment, FBG of ≥ 100 mg/dl or on treatment, TG ≥ of 150 mg/dl or on treatment, and HDL of < 40mg/dl for men and < 50mg/dl for women (10).

#### International Diabetes Federation definition

The IDF definition of MetS is having a WC > 80cm for women and >94 cm for men plus two or more of the following factors: BP ≥ 130/85 mmHg or on treatment, TG of ≥ 150mg/dl or on treatment, HDL of < 40 mg/dl for men and < 50 mg/dl for women or on treatment, and FBG of ≥ 100 mg/dl (10).

#### The harmonized criteria

For the harmonized criteria, MetS is defined as a cluster of three or more of the following five interrelated risk factors: TG of ≥ 150 mg/dl, HDL of < 40 mg/dl for men and < 50mg/dl for women, BP of > 130/85mmHg, FBG of ≥ 100 mg/dl, and a WC of >94 cm for men and >80 cm for women (11).

### Data collection tools and procedures

The standardized questionnaire and document review checklist was prepared in the English language. The data were collected through a self-administered structured questionnaire. Additionally, the patients’ cards were reviewed. Two BSc-trained nurses were selected from hospitals to collect the data and were properly supervised by the principal investigator. The questionnaire was pretested at Asella Teaching Hospital to ensure the quality and consistency of the tool.

### Data processing and analysis

In this study, descriptive statistics of frequency and percentage were used for categorical variables. Mean and standard deviation was used for normally distributed continuous variables. Multivariable logistic regression was performed to analyze the association between MetS and the independent variables. In the adjusted model, when we determined the association between one independent variable and MetS, the effect of the other variables was controlled. All statistical analyses were performed using SPSS software (version: 25.0; SPSS, Chicago, IL) and R software (version: 3.4.3; R Foundation for Statistical Computing, Vienna, Austria). A p-value of < 0.05 was considered statistically significant.

### Ethical consideration

The Ethical Committee that approved this study was the Institutional Review Board of Adama Hospital Medical College. Written informed consent was given by each respondent after the purpose and objectives of the study were explained. Confidentiality and privacy were kept.

## Result

### Socio-demographic characteristics of the participants

A total of 237 participants were included in this study, with a response rate of 95.1%. Of the total, 130 (54.9%) were male. The mean age of the participants was 55 years ( ± 10 SD). Among the study participants, 98 (41.4%) were Orthodox Christians and 120 (50.6%) were of the Oromo ethnicity. More than half of the participants [136 (55.3%)] were urban residents and 160 (67.5%) were married. One-fourth of the study participants [57 (24.1%)] had no formal education. Below half [114 (48.1%)] of the participants earned a monthly income of less than 4230 birrs (**Table 1**).

**Table 1:** Socio-demographic characteristics of T2DM patient at Adama Hospital Medical College, 2022, Adama, Ethiopia (n=237).

Characteristics	Category	Frequency	Percentage
Sex	Male Female	130 107	54.9 45.1
Age	30-50 years 51-70 years >70 years	86 134 17	36.3 56.5 7.2
Religion	Orthodox Muslim Others ^a^	98 75 64	41.4 31.6 27
Ethnicity	Oromo Amhara Others ^b^	120 55 62	50.6 23.2 26.2
Place of residency	Urban Rural	131 106	55.3 44.7
Educational status	No formal education 1-8 grade 9-12 grade Diploma Degree and above	57 58 33 39 50	24.1 24.5 13.9 16.5 21
Monthly per capita income (in birr)	<4230 4230-16500 >16501	114 85 38	48.1 35.9 16
Occupation of respondents	Private Others	108 126	45.6 54.4
Marital status	Single Married Others ^c^	22 160 55	9.3 67.5 23.2

a Protestant, Catholic, Wakefata.

b Tigray, Sidama, Waliata, Silte, Gurage.

c Divorced, Widowed, Separated.

### Medication and health-related factors

Of the study participants, 65 (27.4%) and 70 (29.5%) had a family history of hypertension and diabetes mellitus, respectively. In this study, 85 (35.9%) of the participants had already been diagnosed as having hypertension and 68 (80%) of them are on antihypertensive medication. Approximately one-third of the participants [82 (34.6%)] had a history of dyslipidemia. Out of the study participants, 182 (76.8%) of the patients with T2DM were taking medication (**Table 2**).

**Table 2:** Medication and health-related factors among T2DM patients at Adama Hospital Medical College, 2022, Adama, Ethiopia (n=237).

Variables	Category	Frequency	Percentage
Family history of hypertension disease	Yes No	65 172	27.4 62.6
Family history of DM	Yes No	70 167	29.5 70.5
Types of DM medication	Tablet Insulin Both	182 26 29	76.8 11 12.2
Personal history of hypertension	Yes No	85 152	35.9 64.1
Medication for hypertension	Yes No	68 17	80 20
Personal history of dyslipidemia	Yes No	82 155	34.6 65.4

### Behavioral and dietary-related factors

Regarding smoking status, 175 (73.8%) of the participants never smoked cigarettes, 32 (13.5%) were current smokers, and 30 (12.7%) were former smokers. Only 14 (14.3%) and 41 (17.3%) of the participants were khat chewers and alcohol drinkers in the previous 12 months, respectively. In total, 58 (24.4%) of the study participants performed physical exercise regularly. Similarly, 23 (9.7%) and 8 (3.4%) of the study participants have sleep disorders and psychiatric problems, respectively. Of the total participants, 50 (21%) have a meal plan. Above half [139 (58.5%)] of the participants were vegetable oil users and 127 (53.6%) had a BMI greater than 25kg/m^2^ (**Table 3**).

**Table 3:** Behavioral factors among T2DM patients at Adama Hospital Medical College, 2022, Adama, Ethiopia (n=237).

Variable	Category	Frequency	Percentage
Smoking status	Never smoker Former smoker Current smoker	175 30 32	73.8 12.7 13.5
Khat chewing	Yes No	34 203	14.3 85.7
Alcohol drinking in the past 12 months	Yes No	41 196	17.3 82.7
Amount of alcohol drunk per week	<200 gm 200-400 gm >400 gm	14 18 9	34.1 43.9 22
Physical activity	Yes No	58 179	24.4 75.6
Sleep disorder	Yes No	23 214	9.7 90.3
Adequate sleep	Yes No	197 40	83.1 16.9
Psychiatric illness	Yes No	8 229	3.4 96.6
Meal plan	Yes No	50 187	21 79
Oil type	Vegetable Palm Others ^d^	139 81 17	58.5 34.1 7
BMI	< 25 ≥25	110 127	46.4 53.6

d Suet, Butter.

### The prevalence of MetS

Overall, the prevalence of MetS among patients with T2DM was 53.2%% (95% CI: 46.8 - 59.6), 41.3% (95% CI: 35.0 - 47.5), and 41.8% (95% CI: 35.5 – 48.1) based on the 2009 harmonized, NCEP-ATP III and IDF criteria, respectively. There was substantial agreement between the 2009 harmonized criteria with both the NCEP-ATP III and the IDF criteria (kappa >0.61) (**Table 4**).

**Table 4:** Agreement test between the three criteria of Metabolic syndrome (MetS) among T2DM patients at Adama Hospital Medical College, 2022, Adama, Ethiopia.

Ethiopia		NCEP-ATP III criteria		Kappa
		MetS	no MetS	
**IDF criteria**	MetS	71	28	0.52
	no MetS	27	111	
		**NCEP-ATP III criteria**		
		MetS	no MetS	
**2009 harmonized criteria**	MetS	98	28	**0.76**
	no MetS	0	111	
		**IDF criteria**		
		MetS	no MetS	**0.77**
**2009 harmonized criteria**	MetS	99	27	
	no MetS	0	111	

Bold, indicates significance.

For our final model, we used the 2009 harmonized criteria to define MetS. The value of the kappa test was between 0.6 to 0.8 which indicates a substantial agreement between the two measurements. Therefore, the 2009 harmonized definition criteria were substantially in agreement with both NCEP-ATP III and IDF criteria (kappa >0.61).

### Factors associated with MetS

The crude and adjusted estimates for MetS are shown in **Table 5**. In the adjusted model, among the patients with T2DM, those who were urban residents had increased odds of metabolic syndrome compared with rural residents (AOR=3.07, 95%CI: 1.46-6.42). Earning a monthly income greater than 4230 birrs had a significant association with MetS among the patients with T2DM (P-value<0.05). Having a history of hypertension (AOR =2.65, 95% CI: 1.22-5.78) and dyslipidemia (AOR= 4.47, 95% CI: 1.96-10.19) were associated with increased odds of MetS among the patients with T2DM. Cigarette smokers among the patients with T2DM were 6.2 (AOR=6.2, 95% CI 1.70-22.93) times more likely to develop MetS than a non-smoker. Patients with T2DM who spent their leisure time in sedentary activities such as watching TV and reading a book are more likely to have MetS compared with those who spent their leisure time more actively (AOR=3.62, 95%CI 1.68-7.82). Having no meal plan among the patients with T2DM was significantly associated with metabolic syndrome (AOR=3.97, 95% CI: 1.5-10.48). Using palm oil was significantly associated with MetS compared to vegetable oil (AOR=4.87, 95% CI 2.06-11.51). Patients with T2DM with a BMI greater than or equal to 25 kg/m2 had a significant association with high odds of MetS (AOR=3.97, 95% CI: 1.5-10.48).

**Table 5:** Bivariable and multivariable logistic regressions on the predictors of metabolic syndrome among T2DM patients at Adama Hospital Medical College, 2022, Adama, Ethiopia (n=237).

Variables	Category	Metabolic syndrome		COR (95%CI)	AOR (95% CI)
		Yes (%)	No (%)		
Place of residence	Rural Urban	43(18.2) 83(35.0)	63(26.6) 48(20.2)	1 2.53(1.5-4.280	1 3.07(1.46-6.42) **
Monthly income (in birr)	<4230 4230-16500 16501-51184 >51184 birr	46(19.4) 55(23.2) 23(9.7) 2(0.8)	68(28.7) 30(12.7) 11(4.6) 2(0.85)	1 2.7(1.51-4.84) 3.09(1.37-6.94) 1.4(0.2-10.87)	1 3.62(1.56-8.36) ** 5.87(1.8-19.1) ** 0.99(0.06-15.6)
History of cardiovascular	Yes No	27(11.4) 99(41.8)	38(16) 73(30.8)	1.9(1.07-3.4) 1	3.33(1.41-7.84) 1
History of hypertension	Yes No	57(24) 69(29.2)	28(11.8) 83(35)	2.44(1.4-4.26) 1	2.65(1.22-5.78) ** 1
History of dyslipidemia	Yes No	58(24.4) 68(28.6)	24(10.1) 87(36.7)	3.09(1.74-5.47) 1	4.47(1.96-10.19) *** 1
Smoking status	Never smoker Former smoker Current smoker	81(34.2) 18(7.6) 27(11.4)	94(39.7) 12(5) 5(2.1)	1 1.74(0.79-3.83) 2.3(2.3-7.02)	1 1.39(0.44-4.32) 6.2(1.7-22.93) **
Spend leisure time	Sedentary activity Activity	91(38.4) 35(14.7)	50(21.1) 61(25.8)	3.17(1.84-5.44) 1	3.62(1.68-7.82) *** 1
Meal plan	Yes No	10(4.2) 116(49)	40(16.8) 71(30)	1 6.53(3.07-13.88)	1 **3.97(1.5-10.48**) **
Oil type	Vegetable Palm Others ^d^	63(26.1) 55(23.2) 8(3.3)	76(32.4) 26(10.9) 9(3.7)	1 2.55(1.43-4.52) 1.07(0.39-2.94)	1 4.87(2.06-11.51) *** 1.93(0.45-8.23)
BMI	< 25 ≥25	44(18.6) 82(34.6)	66(27.8) 45(19)	1 2.73(1.61-4.63)	1 3.36(1.57-7.16) ***

Significant code: 0 “***”, 0.001 “**”, 0.01 “*”, ^d^Suet, Butter. Bold, indicates significance.

## Discussion

Overall, the prevalence of metabolic syndrome among patients with T2DM was 53.2%% (95% CI: 46.8 - 59.6), 41.3% (95% CI: 35.0 - 47.5), and 41.8% (95% CI: 35.5 - 48.1) based on the 2009 harmonized, NCEP-ATP III, and IDF criteria, respectively. Our results are comparable with studies conducted in China (50%) and Nigeria (55.7%) which were based on the 2009 harmonized criteria (12, 13). A study conducted in Dessie Referral Hospital suggested that the prevalence of MetS was 50.3%, 59.4%, and 64.5% according to the IDF, NCEP-ATP III, and 2009 harmonized criteria, respectively, which was higher than the finding of this study (14).

In this study, impaired glucose tolerance (IGT) or diabetes, central obesity, and high triglycerides (TGC) are the common components of MetS in women, whereas hypertension and central obesity are the common components in men. While in a study in Dessie, hypertension and central obesity were common in women and hypertension and high TGC were common in men (14). In a Sri Lankan study, hypertension and central obesity were the common components of MetS (15). In addition, a study done in Mekelle suggested that a high TGC and central obesity is the common component of MetS (16). The existence of different criteria used to define MetS among studies may lead to slight discrepancies in the results.

The study shows that, among patients with T2DM, being an urban resident was significantly associated with MetS compared with rural residents. These findings are in line with the findings of previous studies (5, 17). These studies suggested that a sedentary lifestyle (unhealthy dietary practices and lack of exercise) is predominantly high in urban areas. As a result, it is not surprising that urban residents have a high risk of MetS compared with rural residents.

Earning a relatively high income per month was associated with an increased risk of MetS among patients with T2DM compared to low-income-generating individuals. This finding is similar to most of the findings of the studies done in the USA, India, Türkiye, and Ethiopia (18–21). This is because an individual who earns a high income increases the chance of taking excess calories and using more time for a sedentary lifestyle.

According to this study’s findings, a family history of CVD was associated with an increased risk of MetS among patients with T2DM. This finding is in agreement with the finding of a study conducted in Tunisia, in which all the components of MetS and MetS itself were higher among individuals who had a family history of CVD (22). There are different explanations given for the associations. The first is that they are due to a genetic predisposition to components of metabolic syndrome, especially in those with T2DM and hypertension. The second explanation is that the family shares lifestyle factors, such as a risky diet.

In this study, among patients with T2DM, current smokers were significantly associated with MetS. However, in contrast to our findings, studies conducted in Tunisia and Hawassa reported no significant association between cigarette smoking and MetS among patients with T2DM (22, 23). This difference may have happened because, in our study, we classified the smoking status into three: former, current, and never smoker, which was not done in the previous studies. We hope that our classification is proper as it helps to determine the existence of the association.

Spending more leisure time on sedentary activities had a statistically significant association with MetS among patients with T2DM. Patients who spent their leisure time watching television, reading, or other sedentary activities had 3.21 higher odds of MetS compared to those who spent their time walking and cycling. A similar finding was reported in studies conducted in Palestine and Dessie (14, 24). This might be because sedentariness causes obesity, insulin resistance, and poor lipid metabolism.

This study found that using palm oil increased the odds of MetS among patients with T2DM by 4.87. This finding is supported by a study conducted in southwest Ethiopia in which palm oil usage increased the risk of MetS by 5.567 compared to other oil-type users (25). This is because palm oil has high saturated fatty acid which is a risk factor for T2DM, hypertension, and dyslipidemia. In contrast to our findings, a study conducted in Dessie reported palm oil users were less likely to develop MetS (14). We found that being overweight and obese were significantly associated with MetS among patients with T2DM. This finding is similar to the findings from studies conducted in Ghana and Ethiopia (Mekelle and Gondar) (16, 17, 26). In reality, obesity and dyslipidemia are the main risk factors for MetS among patients with T2DM.

The result of this study shows that having a history of hypertension and dyslipidemia increases the risk of MetS among patients with T2DM. To the best of our knowledge, no study has assessed the association between of history of hypertension and dyslipidemia with MetS. Since both are components of MetS criteria, their presence increases the chance of MetS.

There are some limitations in our study. First, observational studies based on a cross-sectional study design cannot determine a causal relationship between associated risk factors and MetS. Second, as this study was a single public hospital-based study the generalization of our results seems tentative since the vast majority of patients with T2DM outside the facility were not investigated. Third, relatively, our sample size was small which may also affect the robustness of our estimation. Further, a large cohort study is warranted to confirm our findings.

## Conclusion

Overall the magnitude of metabolic syndrome among patients with type 2 diabetes mellitus is high based on the 2009 harmonized, NCEP-ATP III, and IDF criteria. The 2009 harmonized criteria are the most sensitive criteria for diagnosing metabolic syndrome. Being an urban resident, having a high income, having a family history of cardiovascular disease, personal history of hypertension and dyslipidemia, current cigarette smoker, sedentary activity, lacking a meal plan, using palm oil, and having a high BMI were positively associated with metabolic syndrome among patients with T2DM. The authors recommend that health providers should take an iterative approach to make patients aware of the factors mentioned above to reduce the level of MetS and its complications in T2DM.

## Data availability statement

The data analyzed in this study are available from the corresponding author upon reasonable request.

## Ethics statement

The Ethical Committee that approved this study was the Institutional Review Board of Adama Hospital Medical College. Written informed consent was taken from each respondent after the purpose and objectives of the study were explained. Confidentiality and privacy were kept.

## Author contributions

Conceptualization, MG and TC; data curation, MG and TC; formal analysis, MG and TC; funding acquisition, MG; project administration, TC; supervision, TC; writing-original draft, MG and TC; writing-review & editing, MG and TC. Both authors contributed to the article and approved the submitted version.
